# Personal protective equipment solution for UK military medical personnel working in an Ebola virus disease treatment unit in Sierra Leone

**DOI:** 10.1016/j.jhin.2017.03.018

**Published:** 2017-05

**Authors:** P. Reidy, T. Fletcher, C. Shieber, J. Shallcross, H. Towler, M. Ping, L. Kenworthy, N. Silman, E. Aarons

**Affiliations:** a Royal Centre for Defence Medicine, Ministry of Defence, Birmingham, UK; b Liverpool School of Tropical Medicine, Liverpool, UK; c Public Health England, Porton Down, Salisbury, UK; d National Ambulance Resilience Unit, Salisbury, UK

**Keywords:** Personal protective equipment (PPE), Ebola, Ebola treatment unit, Visors, gloves, goggles, boots, Disposable PPE, Re-usable PPE

## Abstract

The combination of personal protective equipment (PPE) together with donning and doffing protocols was designed to protect British and Canadian military medical personnel in the Kerry Town Ebola Treatment Unit (ETU) in Sierra Leone. The PPE solution was selected to protect medical staff from infectious risks, notably Ebola virus, and chemical (hypochlorite) exposure. PPE maximized dexterity, enabled personnel to work in hot temperatures for periods of up to 2 h, protected mucosal membranes when doffing outer layers, and minimized potential contamination of the doffing area with infectious material by reducing the requirement to spray PPE with hypochlorite.

The ETU was equipped to allow medical personnel to provide a higher level of care than witnessed in many existing ETUs. This assured personnel working as part of the international response that they would receive as close to Western treatment standards as possible if they were to contract Ebola virus disease (EVD). PPE also enabled clinical interventions that are not seen routinely in West African EVD treatment regimens, whilst providing a robust protective barrier. Competency in using PPE was developed during a nine-day pre-deployment training programme. This allowed over 60 clinical personnel per deployment to practice skills in PPE in a simulated ETU and in classrooms. Overall, the training provided: (i) an evidence base underpinning the PPE solution chosen; (ii) skills in donning and doffing of PPE; (iii) personnel confidence in the selected PPE; and (iv) quantifiable testing of each individual's capability to don PPE, perform tasks and doff PPE safely.

## Introduction

Ebola virus is found in body fluids such as blood, sweat, vomit and diarrhoea of patients in the acute phase of infection [1]. Contact with patients presents a high risk of nosocomial infection to the medical staff involved with their treatment. In the period from August 2014, when the World Health Organization declared the West African outbreak to be an international public health emergency, through to November 2015, 881 healthcare workers (HCWs) contracted Ebola virus disease (EVD), 531 of whom died [2].

Serious gaps in implementing infection prevention and control (IPC) standards were reported in the settings where transmission likely took place or where infected HCWs were employed. Among these, the most frequently reported were deficiencies in administrative, engineering and environmental controls, inappropriate use or lack of personal protective equipment (PPE), defective IPC practice and behaviour, and poor employment conditions and social determinants [3]. Recent studies have also shown that the baseline skill levels of HCWs in PPE removal are poor, and result in contamination and risk to HCWs [4], [5].

The Ministry of Defence (MoD) Kerry Town Ebola Treatment Unit (ETU) was developed to provide the highest possible standards of care, including interventions such as peripheral and central venous catheters, urinary catheters and blood product transfusions. During the period from admission of the first patient in November 2014 to discharge of the last patient in December 2015, the unit admitted 125 patients, 44 of whom had a final diagnosis of EVD. It was important that IPC standard precautions were maintained to reduce the risk of healthcare-associated infections for medical staff and patients.

## Rationale for selecting PPE

In September 2014, specialists from Public Health England, the National Ambulance Resilience Unit and the Ministry of Defence (MoD) worked together to identify the combination of PPE and donning and doffing protocols for PPE worn by military medical personnel working in a 12-bedded ETU in Kerry Town, Sierra Leone. PPE comprised both single-use and re-usable items. It needed to be available in a variety of sizes; to be resistant to heat, sweat and chemicals; to minimise loss of dexterity; and to maximize movement. Furthermore, the items needed to be procured during a time when many countries were seeking to stockpile PPE, making development of a sustainable solution critical to allow for continuity and the establishment of reliable supply.

The rationale behind the PPE solution was to allow for care to be delivered whilst protecting those working within the ETU, providing a barrier against nosocomial infection.

PPE was doffed in a designated area, prior to exit into the green zone [6]. A buddy–buddy system where personnel were consistently partnered with the same individual was established. Personnel checked each other's PPE and practices at each stage of all activities from donning PPE, working in the red zone (clinical), and doffing PPE to provide assurance during clinical work.

## PPE selected

### Scrubs

Cotton scrubs were chosen as the base layer. Consideration was given to disposable scrubs but these were rejected on the basis of comfort, material degradation and additional clinical waste. Cotton scrubs offered a comfortable, absorbent base that could be bleached, laundered and re-used. Due to the risk of malaria, long-sleeved tops would have been preferred as they offer greater protection against mosquito bites; however, these proved to be significantly more difficult to source within the time frames available.

### Footwear

Rubber boots were chosen as the footwear for the clinical zone as they are both chemical and ultraviolet resistant; the boots needed to be decontaminated by soaking in hypochlorite (5000 ppm) for at least 10 min, rinsed in water and left inverted, exposed to direct sunlight, before being transferred to the green zone changing room for re-use. Rubber boots are flexible and comfortable for wearers, and seamless boots were preferred to aid decontamination. White or bright colours (not red) were the preferred option as contamination could be seen easily. The sole and upper area of the boot needed to be resistant to both cuts and punctures. Furthermore, the sole needed to meet EN13287 SRA and SATRA TM144 standards in order to be slip resistant, whilst not having a deep tread which could be difficult to decontaminate. Immersion foot can be caused by wearing damp socks and shoes for less than one day [7]. It was therefore decided that rubber boots would only be worn within the red zone, and removed as soon as the staff were decontaminated.

### Coveralls

Despite the final choice of coverall suits, back fastening gowns were initially considered as part of the overall PPE solution due to their established use for infectious work. Gowns can be removed away from the wearer and may assist with temperature regulation in a warm environment. However, in this circumstance, a continuous suit that extends to cover the head, torso, arms and legs of the wearer was preferred. Coveralls are produced to suit a variety of settings, and water-resistant coveralls made of a breathable material with taped seams were selected to reduce the possibility of liquid penetration and contamination of exposed areas under the coverall.

The single-use coveralls chosen and tested were made of polyethrine and polypreprine (both breathable materials). These materials had a lower thermodynamic specification compared with other PPE available on the market, which potentially allowed for a longer working time in the red zone. Coveralls with finger loops were selected as these anchored the sleeve and prevented it from sliding up. Loops were worn on the middle finger as this minimized loss of dexterity. Integrated booties could potentially cause a trip hazard so were not preferred.

### Apron

Aprons were included within the PPE solution to increase protection to the front of the wearer, as this area was considered to be at high risk of splashes/spills of contaminated material and, in addition, the coverall zip was set into permeable material. The properties stipulated were: length (below knee), plastic and lightweight design (minimum 16-μm thickness, so it would stay in place but could be torn off deliberately as part of the removal process), fluid repellent and disposable. The apron chosen was adjustable, and so could cover the zip completely, irrespective of body shape, and helped to minimize heat stress whilst giving the necessary protection. The recommendation was to change aprons and gloves between patients in order to reduce the risk of cross-contamination between patients.

### Gloves

From the outset, double gloving was the agreed standard practice to allow outer gloves to be changed between patients whilst still protecting the HCW. Tactility and dexterity through two pairs of gloves was of key importance. In addition to complying with European standard EN 374-2:2003 for resistance to penetration by chemicals and micro-organisms, avoidance of allergic reactions was considered from both a patient and wearer perspective. These factors led to the choice of 400-mm nitrile, powder-free gloves.These long gloves gave additional coverage to the arm, coupled with a secure elasticized coverall fitting around the wrist; this, together with the coveralls' attached finger loops, held the coverall cuffs in place and minimized the risk of exposure of the hands, wrists and lower arms. The first pair was worn under the cuff of the coverall, with the second pair worn over the cuff and extending up the arm. The guidance of the US Centers for Disease Control and Prevention [8] described the issues that may have been faced if the gloves were taped to the sleeves of the coverall; therefore, this practice was decided against in order to eliminate these potential problems when doffing.

### Face mask and visor

The use of goggles was initially considered but, following assessment of the environment and the level of care that would be delivered, it was decided that goggles would be too restrictive and the vision range would be too poor.

A disposable filtered face piece 2 (FFP2), non-valved mask was selected to be worn under a face visor. Although the Ebola virus is not spread via the airborne route, it was essential to protect the eyes, mouths and noses of HCWs from droplets and splashes. An FFP2 mask in combination with a full face visor was considered to be sufficient to control the risk of fluid splash. Non-valved or shrouded valve FFP2 masks were preferred as these offer a greater level of protection from splashes. The masks chosen were suitable for use in hot and humid conditions, and were compatible with the face visors. Additionally, the shape of the masks and the way they were donned made the removal process safe.

Two types of multi-use adjustable visors were chosen as these could fit the diversity of personnel deployed comfortably and securely. Repeated decontamination of visors in high levels of hypochlorite solution led to delamination and clouding of the visors and rusting of metal supports, so both types of visors had replaceable acetate screens and plastic adjustable head pieces. After 10 min of soaking in 5000 ppm hypochlorite, the visors were rinsed in water, dried and returned for re-use. The visors provided good head movement and a greater vision range, thereby increasing situational awareness. A clear full face visor also meant that patients could see the clinician's face, allowing better communication and reassurance. Staff reported that the visors reduced the feelings of claustrophobia, and allowed air movement and a cooling effect.

### Headwear

Two layers of disposable head wear were worn: a surgical cap, to aid with coverage of the front of the head and prevent hair falling in front of an individual's eyes; and the hood of the coverall suit. The hood of the coverall suit was worn over the upper edge of the visor, allowing the hood to be removed prior to the visor in the doffing stage, thus maintaining protection of the mucosal membranes for longer during the doffing procedure. Fluid-repellent caps were preferred; however, surgical caps of several styles were found to be suitable. Most clinical PPE were single-use items, apart from scrubs, face visors and boots. Re-usable items were cleaned and decontaminated by the wash team prior to re-use.

## Procedural rationale

### Donning and doffing

Personnel suffering from stress, fatigue, heat degradation and dehydration are at a higher risk of making errors [9]. PPE monitors were used to direct the doffing process. Their role was to ensure that drills were carried out correctly, in a calm, controlled manner, to assist doffers if required, manage doffing errors and PPE breaches, and therefore minimize the risk of contamination and stress to the individual.

### Donning

**Figure 1:**
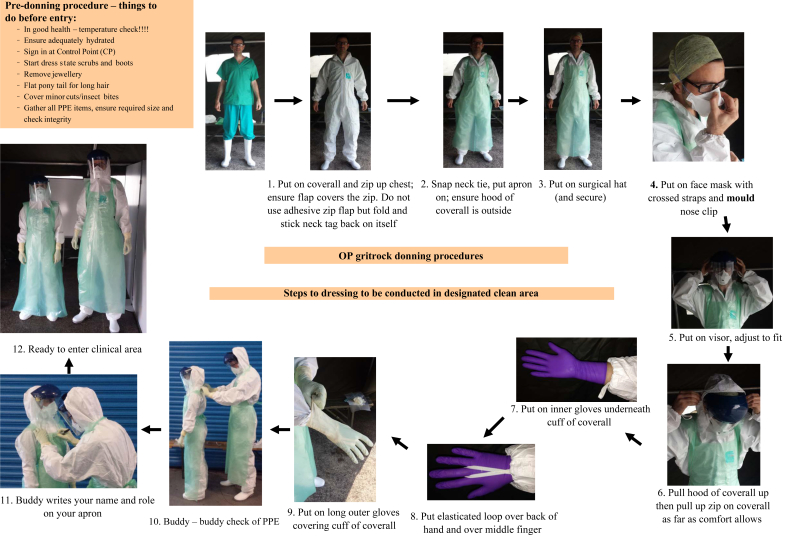
Donning steps conducted in a buddy–buddy system. PPE, personal protective equipment.

The donning and doffing procedures were adapted from the systems already in use, and were familiar to those used by ambulance hazardous area response teams when wearing the civilian responder (CR1) suit. Whilst this equipment was unfamiliar to military personnel, similar procedures are used with the military Mark 1V chemical biological radiological and nuclear protection suit, and the training reflected this. A stepwise approach (Figure 1) was developed to ensure that no items of PPE were missed out, and allowed buddy–buddy checking of integrity and fit.

### Glove and apron changes between patients in the red zone

Personnel decontaminated their gloves (with hypochlorite solution 5000 ppm, using handwashing procedure) and then changed their gloves and aprons between tasks and patients, as per UK best practice [10], [11] and military medical policy. This was undertaken using the buddy–buddy system to ensure that no steps were missed out and the correct sequence was followed.

### Doffing

**Figure 2:**
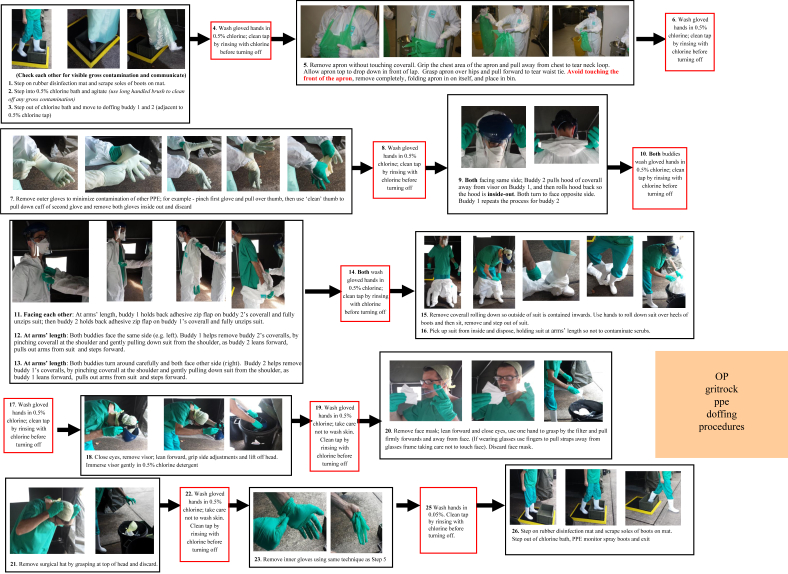
Doffing steps were always under the instruction of personal protective equipment (PPE) monitors. The mask shape made it easy to get a safe secure grip on the front for doffing, thereby avoiding contamination of the hair with either Ebola or bleach. Personnel pinched the top front of the apron after washing hands to tear the apron off safely; this area was least contaminated. Hands were washed afterwards.

The doffing procedure worked on a stepwise approach under instruction from PPE monitors (Figure 2). This ensured minimal risk of contamination to the wearer by reducing the chance of contaminating lower layers of PPE and skin, and reducing contamination of the environment by zoning the doffing area. HCWs doffed in pairs so that they could see where they were touching at all times. The order of removal maximized protection of mucosal membranes and minimized exposure to chlorine hazards. Similar methods have been used by the military and ambulance hazardous area response teams for a number of years, and were adapted to fit the chosen PPE. Between removal of each item of PPE, the wearer was required to decontaminate their hands with hypochlorite solution (5000 ppm) to prevent recontamination of PPE.

## Training and monitoring

Training was delivered in a graduated manner in line with MoD practices; this aided familiarity for the personnel, allowed them to understand the process and adopt it safely [12], and understand the evidence base behind the equipment. Following the formal presentation, personnel were shown videos demonstrating the donning and doffing protocols which had been filmed with MoD personnel under the guidance of subject matter experts. This promoted familiarity with the techniques prior to personnel undertaking hands-on training. Participants were split into small groups with instructors to practice and clarify key points. The training culminated in a simulated ETU environment in which personnel used PPE protocols whilst treating mock patients: ultraviolet tracer dye was used to simulate bodily fluids contaminated with the Ebola virus. During this exercise, the personnel were monitored by experts from the contributing agencies using closed-circuit television and body-mapping techniques to identify contamination of PPE and the ward area, demonstrating the effectiveness of cleaning and doffing protocols, and providing further support for validation of PPE and protocols. Of 312 body-mapping procedures recorded after removal of PPE and decontamination, there were only four instances of contamination; these were restricted to the soles of rubber boots [13]. This was eliminated by modification of the cleaning/decontamination of boots.

Refresher training was given to staff *in situ* and at regular intervals to ensure that they were confident and competent with the protocols once in West Africa. This continued in order to maintain competence until the outbreak was declared over and the facility closed down.

## MoD Kerry Town ETU

The ETU concept and doctrine that was developed was designed to offer up to high-dependency level care for HCWs in a resource-limited West African setting. It was well resourced with medical and nursing staff, with a nurse:patient ratio of 1:1–1:2 depending on occupancy levels. For each 60-day clinical deployment period, up to 50 HCWs provided clinical care in the red zone. The initial 12 beds (later increased to 20-bed capacity) provided a suspected EVD area, with four single-occupancy rooms with dedicated individual toilets. There was no cohorting of suspected EVD patients and no cases of nosocomial transmission to suspected EVD patients. The tents and surrounding areas had closed-circuit television to allow remote monitoring of staff and patients, and were temperature controlled.

## Results

The PPE solution described for the MoD-led ETU at Kerry Town was used by personnel deployed with the 22^nd^ Field Hospital as part of Operation GRITROCK between November 2014 and June 2015. A large number of personnel used PPE, and the combination of equipment chosen could be adapted to fit all the deploying personnel.

Lamb *et al*. [6] summarized how the MoD considered Operation GRITROCK to be a success due to ‘excellent clinical care, validated through the WHO [World Health Organization] inspection…and a far lower than expected EVD infection rate among HCWs’. Medical workers were protected by the combination of PPE, donning and doffing procedures, and working practices used within the facility. The conditions within the facility were harsh; temperatures and humidity were high, the facility was constructed rapidly with materials available locally, and these factors had implications for the use of PPE in this clinical setting.

Temperatures in Sierra Leone typically reached 31 °C, which caused people wearing PPE to perspire heavily. Some batches of gloves degraded on contact with perspiration, visibility could be reduced by condensation on the visors, and sweat could pool in the rubber boots. This was managed by limiting the amount of time that staff spent in the red zone to a maximum of 2 h. There was a protocol for heat stress and emergencies in the red zone, allowing safe extraction of staff should the need arise.

The quality of PPE was monitored and any failures were managed using a formal reporting mechanism. Issues with insufficient thickness of aprons, delamination of visor screens, perspiration-sensitive gloves and variability between batches of coveralls were identified and addressed in this way. Faulty batches of PPE were disposed of and replacements were sourced quickly.

Observed breaches in PPE included rips in coveralls caused by catching on door handles or rubbing against rough wooden balustrades. This was mitigated by removing door handles where possible, and wrapping the balustrades in cushioned material. Other breaches in PPE included the coverall hoods or headwear slipping off within the red zone. The headwear protected the user from chlorine dripping off the tent flaps, and contact from patients who occasionally patted medical staff on the head as they leant over. If the headwear was displaced, the wearer proceeded to the doffing station immediately, but the risk of contracting Ebola from this was considered low and so did not result in quarantining of the individual.

PPE was designed to protect the wearer primarily from infectious risks, but also from the high concentrations of hypochlorite used. These levels caused the re-usable PPE (boots and visors) to disintegrate and glaze/delaminate, respectively. Rinsing in fresh water and wiping followed by air drying helped to reduce the degradation of this equipment. During the deployment, one individual contracted EVD via an unknown route. As discussed in the paper by Lamb *et al*. [6]: ‘a case review of the events leading up to this case could find no obvious problem with their PPE use, but as a precaution the duration any HCW could remain in the red zone was reduced to 90 minutes’.

Furthermore, two individuals undertook precautionary medical evacuation following needlestick incidents which, when investigated, were not attributed to a failure in PPE. PPE was not designed to protect the wearer from sharps, and therefore strict protocols were used to protect against this hazard.

The robust military management style helped to ensure that donning and doffing drills were followed accurately and consistently. This was further enforced by the role of PPE monitors who ensured that the doffing area was well managed, and instructed and observed each person through the doffing procedure and checked for any breaches in PPE. During the deployment, small, authorized changes were made to the protocols defined pre-deployment in order to speed up the doffing process whilst not compromising IPC, and the protocols described here are those used and tested in Sierra Leone.

## Discussion

Developing a PPE solution for those deploying to the ETU was a critical stage in the UK MoD response because PPE, protocols and the use of disinfectant were the major infection prevention controls. The personnel who would be manning the treatment centre had to be assured and confident in the selection of equipment and the training provided as the situation facing them was unique and not comparable to other military deployments. The use of ultraviolet tracer during training revealed no cross-contamination, unlike recent reports by Casanova *et al*. [4] and Tomas *et al*. [5] which suggested ongoing contamination due to incorrect doffing procedures.

A range of subject matter experts, including experienced emergency responders, and clinical, scientific, laboratory and military staff, worked together to develop and deliver an intense nine-day training course that encompassed the predicted environment that would be faced during deployment. When transferred to the field environment, initial training could then be supplemented and any issues faced fed back to the UK team, allowing for amendments to be made to protocols if required. This continuous feedback loop ensured that the most up-to-date protocols were used for training, and provided a quality control aspect.

The PPE solution chosen was robust and implemented successfully within the ETU in Sierra Leone. However, limitations in the PPE selected have been described, and the authors would lobby for further improvements in PPE design. Such improvements would lead to decreased heat stress for the user and a more simple doffing solution, whilst not compromising infection control methods and maintaining protection from direct and indirect contamination.

## Conflict of interest statement

None declared.

## Funding sources

No specific funding. TF is funded by the Wellcome Trust (104480/Z/14/Z) and the UK MoD.
